# Association of Porcine Swine Leukocyte Antigen (SLA) Haplotypes with B- and T-Cell Immune Response to Foot-and-Mouth Disease Virus (FMDV) Peptides

**DOI:** 10.3390/vaccines8030513

**Published:** 2020-09-08

**Authors:** Patricia de León, Rodrigo Cañas-Arranz, Yago Saez, Mar Forner, Sira Defaus, Dolores Cuadra, María J. Bustos, Elisa Torres, David Andreu, Esther Blanco, Francisco Sobrino, Sabine E. Hammer

**Affiliations:** 1 Centro de Biología Molecular “Severo Ochoa” (CSIC-UAM), 28049 Madrid, Spain; pdeleon@cbm.csic.es (P.d.L.); rcannas@cbm.csic.es (R.C.-A.); mjbustos@cbm.csic.es (M.J.B.); elisa.torres@inia.es (E.T.); 2 Computer Science Department, Universidad Carlos III of Madrid, Leganés, 28911 Madrid, Spain; yago.saez@uc3m.es; 3 Departament de Ciències Experimentals i de la Salut, Universitat Pompeu Fabra, 08003 Barcelona, Spain; mar.forner@upf.edu (M.F.); sira.defaus@upf.edu (S.D.); david.andreu@upf.edu (D.A.); 4 Computer Science Department, Universidad Rey Juan Carlos, 28933 Móstoles, Spain; dolores.cuadra@urjc.es; 5 Centro de Investigación en Sanidad Animal (CISA-INIA), Valdeolmos, 28130 Madrid, Spain; blanco@inia.es; 6 Institute of Immunology, Department of Pathobiology, University of Veterinary Medicine Vienna, A-1210 Vienna, Austria

**Keywords:** swine, foot-and-mouth disease virus, dendrimer peptide, swine leukocyte antigen, SLA polymorphism, adaptive immunity, vaccine, protection

## Abstract

Dendrimer peptides are promising vaccine candidates against the foot-and-mouth disease virus (FMDV). Several B-cell epitope (B_2_T) dendrimers, harboring a major FMDV antigenic B-cell site in VP1 protein, are covalently linked to heterotypic T-cell epitopes from 3A and/or 3D proteins, and elicited consistent levels of neutralizing antibodies and IFN-γ-producing cells in pigs. To address the contribution of the highly polymorphic nature of the porcine MHC (SLA, swine leukocyte antigen) on the immunogenicity of B_2_T dendrimers, low-resolution (Lr) haplotyping was performed. We looked for possible correlations between particular Lr haplotypes with neutralizing antibody and T-cell responses induced by B_2_T peptides. In this study, 63 pigs immunized with B_2_T dendrimers and 10 non-immunized (control) animals are analyzed. The results reveal a robust significant correlation between SLA class-II Lr haplotypes and the T-cell response. Similar correlations of T-cell response with SLA class-I Lr haplotypes, and between B-cell antibody response and SLA class-I and SLA class-II Lr haplotypes, were only found when the sample was reduced to animals with Lr haplotypes represented more than once. These results support the contribution of SLA class-II restricted T-cells to the magnitude of the T-cell response and to the antibody response evoked by the B_2_T dendrimers, being of potential value for peptide vaccine design against FMDV.

## 1. Introduction

The adaptive immune system comprises two arms—one responsible for the antibody (B-cell), the other for the cytotoxic (CTL) response—and both dependent on T-helper cells (Th cells). Presentation of T-cell epitopes in association with MHC class I and class II molecules is the initial and essential step in the activation of T CD4^+^ and CD8^+^ responses. Accordingly, an efficient vaccine must incorporate epitopes recognized by both B- and T-cells, ideally with the latter being widely recognized and presented by MHC alleles frequently occurring in individuals from natural host populations [1]. This requirement is particularly relevant for subunit vaccines harboring a limited number of T-cell epitopes for which the chances to be efficiently presented by all MHC haplotypes within a population can result in the existence of ‘‘non- or poor-responder” individuals [2,3].

Foot-and-mouth disease virus (FMDV) is the etiological agent of a devastating livestock disease of great concern worldwide [4,5]. The measures used to control the disease include extensive and frequent vaccination with whole virus inactivated vaccines and are, thus, of major economic importance [6]. Despite conferring FMD protection when adequately formulated, produced, and delivered, inactivated vaccines have limitations: (i) the requirement of a constant cold-chain (4 °C) to preserve the thermal stability of FMDV particles whose immunogenicity decreases significantly, due to its disassociation at higher temperatures into pentamers [7]; (ii) BSL-3 facilities are needed for the production of live viruses [7]; (iii) the problems associated with ensuring the DIVA (differentiating infected from vaccinated animals) condition of vaccines is met [7].

For these and other reasons, the development of alternative vaccines (such as those based on either the viral capsid protein, VP1 [8], or synthetic peptides) are equivalent to specific regions of the same protein have been pursued for decades. Bittle et al. showed that a peptide corresponding to the G-H loop in VP1 capsid protein-induced neutralizing antibodies (nAbs) in mice and protection in guinea pigs [9]. This peptide linearly juxtaposed to the C-terminal of VP1, previously reported to induce nAbs [10], was able to protect cattle from virus challenge [11]. Despite the vaccine potential of FMDV peptides, the main problem faced during decades was their weaker immunogenicity when compared with conventional, inactivated vaccines [12]. Advantages of peptide vaccines include: (i) safety, as a non-infectious material is required; (ii) DIVA condition; (iii) easy handling and storage (no cold chain is required); (iv) chemical stability; and (v) large affordable scale production. These potential benefits led to further attempts to improve their immunogenicity, such as the inclusion of promiscuous T-cell epitopes (recognized by different MHC molecules, capable of evoking adequate T-cell responses) [13]. Indeed, the improvement of epitopic vaccines by incorporation of T-cell epitopes has been reported for several virus models, such as human immunodeficiency virus (HIV) and hepatitis C virus (HCV) [14,15].

We previously reported the association between a polymorphism in bovine MHC (BoLA) class II molecules and both the immune response and protection induced by vaccines based on linear peptides corresponding to the VP1 G-H loop epitope, either alone or in combination with other B-cell epitopes and/or with a VP1 T-cell epitope identified in vaccinated cattle [13]. Protection showed a limited correlation with specific serum neutralizing activity and a higher correlation with the induction of T-cell responses [16]. Consistently, associations were observed between certain DRB alleles (DRB3.2 *1, 3, and 7) and increased levels of protection and the presence of others (DRB3.2 *12 and 18) and lack of protection [16]. These results supported that the gene polymorphisms in bovine class II MHC influenced the recognition of the individual epitopes, resulting in the animal-to-animal variation observed in both humoral and cellular immune responses [2]. In addition, several FMDV T-cell epitopes capable of binding defined swine leukocyte antigen (SLA) class I [17,18] and class II molecules [19] have been described. These results reinforced the need for adequate T-cell activation for efficient peptide-based protection.

On the other hand, epitope multimerization has also been explored to improve the generally low immunogenicity of peptides. One of these multimerization approaches makes use of multiple antigenic peptides (MAPs), in which the molecular scaffold branches out from a lysine core giving rise to a multiple dendrimer display of epitope peptides [20]. One of such dendrimer peptides (B_4_T), harboring four copies of the VP1 G-H loop linked to highly conserved T-cell epitope (T-3A) from FMDV 3A protein [3A (21–35)] [21], was able to protect pigs against homologous FMDV challenge [22]. Remarkably, downsized versions bearing two copies of the B-cell epitope (B_2_T) linked to T-3A (B_2_T-3A) and/or T-3D [B_2_T-3D, another T-cell epitope identified in swine at 3D protein, 3D (56–70) [23], elicited high titers of both, neutralizing antibodies and IFN-γ-expressing cells in this important species [24,25,26]. Other dendrimeric constructions in which two B_2_T molecules were fused tail-to-tail (B_2_T-TB_2_) or in which T-cell epitopes T-3A and T-3D were combined in a single B_2_T platform in the two possible orientations (B_2_TT-3A3D and B_2_TT-3D3A), elicited high levels of neutralizing antibodies and activated T-cells when tested in pigs ([27,28]; P. De León, unpublished data). All these constructions are here generically termed “B_2_T dendrimers”.

The highly polymorphic nature of the porcine MHC, which codes for the swine leukocyte antigens (SLAs), allows for the presentation of a wide panel of antigenic peptides, and thus, influences disease resistance and vaccine responsiveness. As pathogen effects on SLA gene expression drive the regulation of swine immune responses, SLA-typed pigs are used in vaccine design (reviewed in [29,30]). Despite the ongoing domestication process involving selection for favorable traits and inbreeding, pigs still appear to maintain a high degree of SLA diversity as demonstrated by the presence of the 266 and 227 class I and class II alleles, respectively [29]. To date, there are 73 independent class I (*SLA-1*, *-2*, *-3*) and 51 class II (*DRB1*, *DQB1*, *DQA*) assigned haplotypes [29].

In the set of experiments in which the immunogenicity of B_2_T dendrimers was assessed, a total of 73 peptide-immunized and control animals were analyzed. In these experiments, the porcine T-cell epitopes selected to be part of the B_2_T dendrimeric vaccines evoked T-cell responses in all the 63 peptide-immunized pigs analyzed, being these responses high in most of the animals, which is in line with the high titers of neutralizing antibodies that, in average, they elicited.

In this study, we have typed these pigs at the SLA-I (*SLA-1*, *SLA-3*, *SLA-2*) and SLA-II (*DRB-1*, *DQB-1*, *DQA*) loci and analyzed the associations/correlations between particular SLA low-resolution haplotypes (Lr-Hp) with neutralizing antibody and T-cell responses induced by B_2_T peptides.

## 2. Materials and Methods

### 2.1. Peptide Vaccines

Peptides identified as B- and T-cell epitopes of FMDV O/UKG/11/01 [21,23,24] were built into dendrimer constructs by means of (i) a B-cell epitope (VP1 residues 140–158; PVTNVRGDLQVLAQKAART) plus a C-terminal Cys residue (free thiol form). (ii) Using T-cell epitope, 3A (3A residues 21–35; AAIEFFEGMVHDSIK) and/or 3D (3D residues 56–70; IFSKHRGDTKMSAED) elongated at the N-terminal with two Lys residues followed by an additional Lys branching derivatized as two maleimide groups, as described [22,31] (Table 1).

### 2.2. Pigs and Experimental Design

The immune response to B_2_T dendrimer peptides was assessed in 9 to 12 weeks-old White cross-bred Landrace female pigs (Agropardal SA breed) for experiments 1, 3, 4, and 5 and TOPIGS 20TM breed for experiment 2 (Table 2). Pigs were randomly assigned to different animal groups and immunized by intramuscular injection with 2 mL of Montanide ISA 50V2 emulsion (Seppic, Puteaux, France) with the B_2_T dendrimer peptide as the schedule shown in Table 2. Non-vaccinated pigs inoculated with PBS with Montanide ISA 50V2 were included as controls. FMDV neutralizing antibodies to O/UKG/11/01 expressed as virus neutralization titers (VNT) and the frequency of in vitro IFN-γ-producing specific T-cells were determined as described [24]. For the virus neutralization test, the numbers represent end-point titers, calculated as the reciprocal of the final serum dilution that neutralized 100 TCID_50_ of homologous FMDV in 50% of the wells. The frequency of peptide-specific T cells in the responding population determined by IFN-γ-ELISPOT is expressed as the mean number of spot-forming cells/10^6^ PBMCs, with background values (number of spots in negative control wells) subtracted from the respective counts of stimulated cells.

### 2.3. SLA Typing by PCR-SSP

SLA polymorphisms were analyzed using DNA extracted from PBMC of immunized animals. Therefore, genomic DNA was isolated from porcine PBMC using a DNeasy Blood and Tissue Kit (Qiagen, Düsseldorf, Germany) according to the manufacturer’s instructions. SLA class I (SLA-I) and SLA class II (SLA-II) low-resolution haplotypes (Lr-Hp) were identified by a PCR-based typing assay (PCR-SSP) to define the animals’ MHC background on the allele-group level. SLA typing was performed by PCR with the complete set of typing primers specific for the allele groups of three SLA class I loci, *SLA-1*, *SLA-2*, and *SLA-3*; and three SLA class II loci, *DRB1*, *DQB1*, and *DQA* [32,33].

The criteria and nomenclature used for SLA-I and SLA-II haplotyping were based on those proposed by the SLA Nomenclature Committee [34] and reviewed in [29]. Interpretation of the results was based on the presence of allele-specific PCR products of the expected size in each lane. Low-resolution SLA class I and class II haplotypes were deduced based on the comparison with previously published haplotypes [34] and reviewed in [29] and unpublished breed or farm-specific haplotypes (S.E. Hammer, C.-S. Ho, unpublished data).

### 2.4. Data Preparation and Statistical Analyses

Before conducting the analysis, the two haplotypes (determined for both SLA-I and SLA-II, respectively) were combined and converted to a text string in which the order in the combination was irrelevant (X + Y ≥ XY) and (Y + X ≥ XY). On the other hand, since both T-cell responses and antibody titers were continuous variables, they were discretized in 3 (0: <50; 1: >50 and <90; 2: >90 and <140; 3: >140) and 5 (0: <0.0; 1: >0.9 and <1.5; 2: >1.5 and <1.8; 3: >1.8 and <2.4; 4: >2.4 and <3; 5: >3) groups, respectively. Due to the variability in the T-cell response (measured in IFN-γ ELISPOT assays), discretization in the three selected groups was chosen as it distinguishes between low, medium, and high responder animals. The five groups in which the VNTs were discretized corresponded to the titers from each of the ten serum dilutions analyzed (two-fold dilutions starting at ¼). Each value of discretization (from 0–5) included titers from two consecutive serum dilutions. As a robustness analysis, in addition to the proposed discretization, we have conducted alternative correlation tests with different categorizations using a ±4% variations of the proposed limits.

Since SLA-I, SLA-II, and SLA I+SLA II Lr haplotypes data can be considered nominal variables, a non-parametric statistical test, the Chi-square (χ^2^) correlation analysis was used [35]. In addition, the Cramer’s value (Cv) was determined; it does not provide estimates of significance, but measures the strength of the association among variables [36]. This value ranges from 0 to 1, with those closest to one denoting a strong influence among studied variables. To complete the statistical analysis, two additional tests were used: Maximum likelihood ratio chi-square (LR χ^2^) and Fisher’s exact test, more suitable for too small sample size, and analyzing paired variables with less 20-dimension, respectively. In all cases, the null hypothesis was rejected with a confidence greater than 95% (α = 0.05).

### 2.5. Ethics Statement

Experiments 1, 3, 4, and 5: Experimental procedures were conducted in accordance with protocols approved by the CSIC Committees on Ethical and Animal Welfare and by the National Committee on Ethics and Animal Welfare (PROEX 034/15).

Experiment 2: The study was approved (no. 2013121) by the Central Veterinary Institute (CVI) animal experiment ethical review committee in compliance with Dutch law.

## 3. Results

### 3.1. SLA-I and SLA-II Allele-Groups and Haplotypes Identified in the Studied Pigs

A total of 63 samples from pigs immunized with one or two doses of different FMDV dendrimer peptides and 10 samples from control non-immunized animals were available from five previous independent experiments in which pigs were immunized with B_2_T dendrimer peptides (Table 1 and Table 2). These samples were subjected to SLA class I and class II typing, and the allele-groups and the low-resolution haplotypes identified are presented in Table S1.

**Figure 1:**
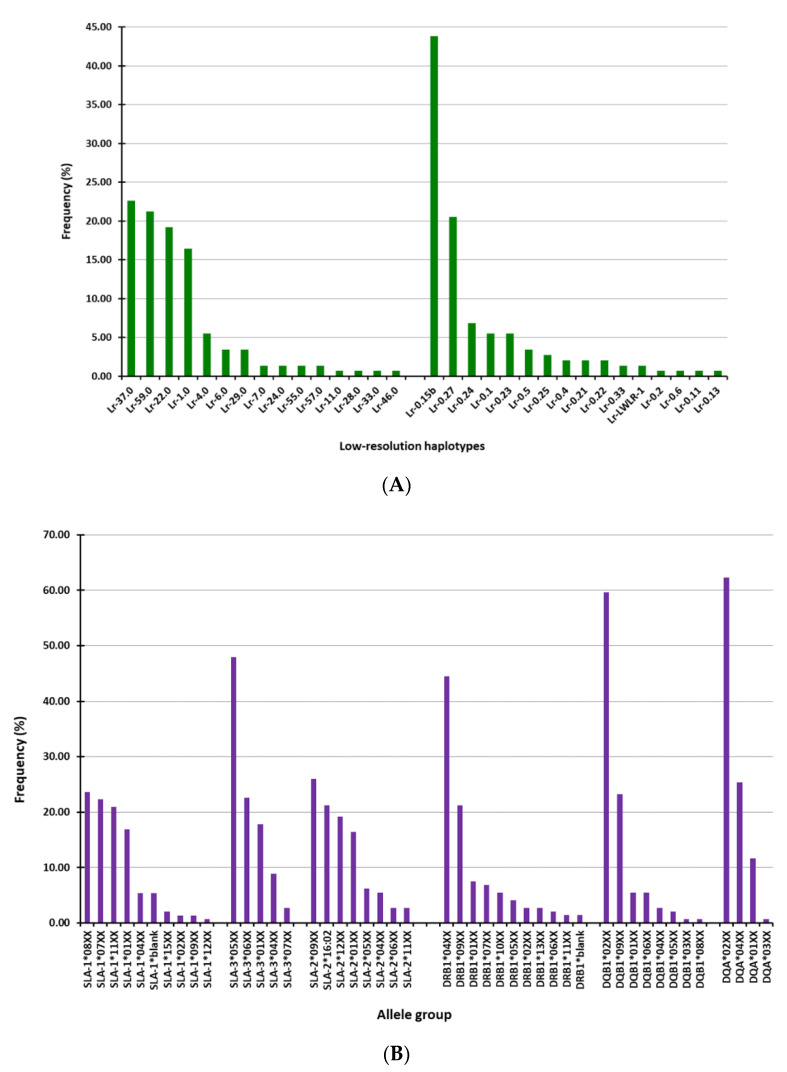
Frequencies of swine leukocyte antigen (SLA)-I and SLA-II allele-groups and Low-resolution (Lr) haplotypes in the cohort of 73 studied pigs. (**A**) Allele-groups at three SLA class I loci (SLA-1, SLA-2, and SLA-3) and three SLA class II loci (DRB-1, DQB-1, DQA). (**B**) SLA-I and SLA-II Lr-haplotypes (Table S1).

In the studied cohort, 23 allele-groups at three SLA class I loci (10 for SLA-1, 5 for SLA-3, and 8 for SLA-2) were identified in the cohort of pigs (Figure 1A), comprising 15 haplotypes (Figure 1B). Likewise, 23 SLA class II allele-groups were found at the three loci analyzed (11 for DRB1, 8 for DQB1, and 4 for DQA), giving rise to 16 haplotypes (Figure 1A,B). Four and two of the Lr haplotypes found in the pigs accounted for more the 90% of the occurrences in class I and class II, respectively (Figure 1B). Accordingly, a higher frequency of certain allele groups was observed in each SLA class I loci (SLA-1*08XX, 07XX, 11XX and 01XX for SLA-1; SLA-305XX, 06XX and 01XX for SLA-3 and SLA-209XX, *16:02, 12XX and 01XX for SLA-2) and SLA class II loci (DRB104XX and 09XX for DRB1; DQB102XX and 09XX for DQB1 and DQA02XX and *04XX for DQA) (Figure 1A). Furthermore, four Lr haplotypes were more frequently observed for SLA class I (Lr-37.0, Lr-59.0, Lr-22.0, and Lr-1.0) and two for SLA class II (Lr-0.15b and Lr-0.27) (Figure 1B).

### 3.2. Correlations between SLA-I and SLA-II Haplotypes and Immune Response to Dendrimer Peptides

**Figure 2:**
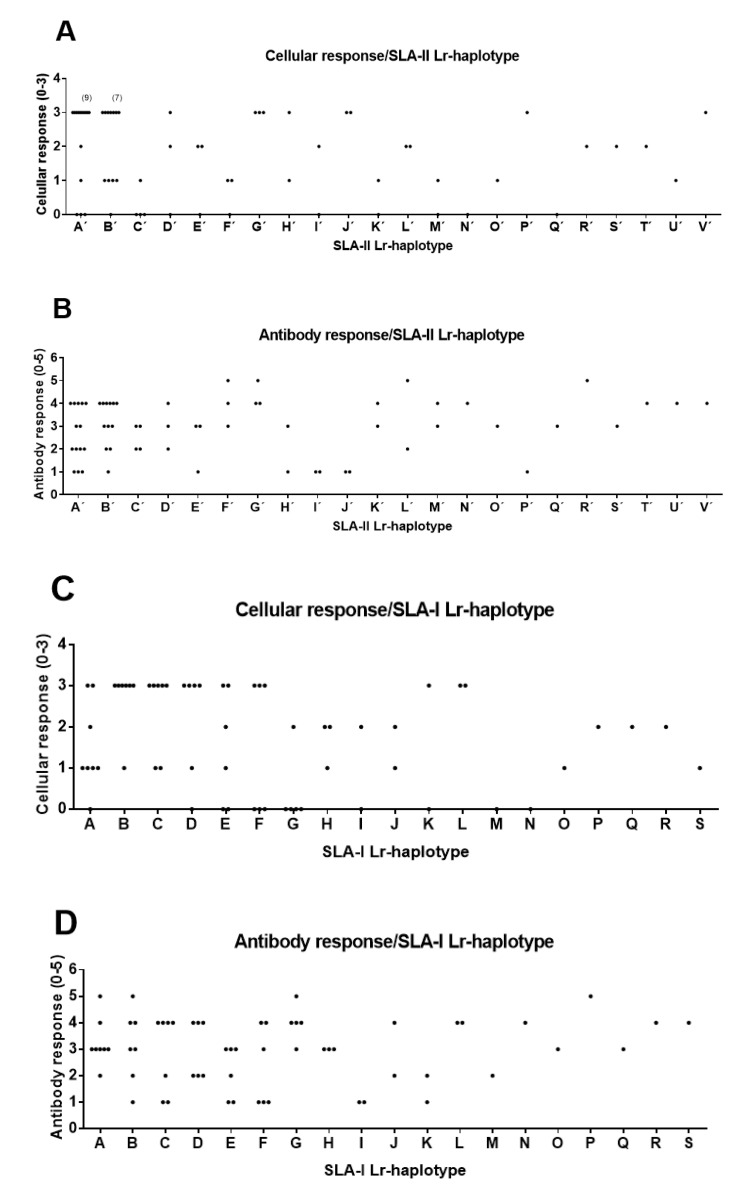
Correlations between SLA-I and SLA-II Low-resolution (Lr) haplotypes and the immune response elicited by dendrimer peptides in the 63 immunized animals (Group 0). Animal group composition as in Table S3. Antibody and cellular response values as in Table S2 and Figure S1. (**A**) Significant correlation of SLA-II Lr-haplotype and cellular response (χ^2^ α = 0.015 *, likelihood ratio chi-square (LR χ^2^) α = 0.038 *, Cv = 0.66). (**B**) No correlation of SLA-II Lr-haplotype and antibody response (χ^2^ α = 0.102, LR χ^2^ α = 0.634, Cv = 0.60). (**C**) Partially significant correlation of SLA-I Lr-haplotype and cellular response. (χ^2^ α = 0.053, LR χ^2^ α = 0.046 *, Cv = 0.59). (**D**) No correlation of SLA-I Lr-haplotype and antibody response (χ^2^ α = 0.065, LR χ^2^ α = 0.251, Cv = 0.57). VNT were categorized as 0: <0.0; 1: >0.9 and <1.5; 2: >1.5 and <1.8; 3: >1.8 and <2.4; 4: >2.4 and <3; 5: >3. IFN-γ secreting T cell values were categorized as: 0: <50; 1: >50 and <90; 2: >90 and <140; 3: >140.

This set of data was used to seek for possible correlations between SLA-I, SLA-II, and SLAI+SLAII Lr haplotypes and the two immune parameters analyzed. To this end, the individual values of VNT (henceforth antibody response), as well as of frequencies of IFN-γ-releasing activated T-cells (henceforth T-cell response) were grouped in five or three categories, respectively (Table S2). In addition, the SLA-I and SLA-II Lr haplotypes defined by the different allele combinations were identified and termed alphabetically (Table 3). Statistical analyses were performed in order to find possible correlations between haplotypes an immune response, and only those correlations giving positive values with χ^2^ and LR χ^2^ tests (α < 0.05), were considered as significant. Indeed, the only robust significant correlation found for the 63 B_2_T immunized pigs (group 0) was noticed between SLA-II Lr haplotype and the T-cell response (Figure 2A), which was statistically significant when using the χ^2^ (α = 0.015) and LR χ^2^ (α = 0.038) tests, and with a Cramer’s value of Cv = 0.66, which indicates a strong association among variables (Table 4). In addition, all correlation tests conducted with alternative discretization limits (±4%) were also statistically significant for SLA-II Lr haplotype and the T-cell response, supporting the evidence of having a strong and robust association among these variables. A trend for the association of SLA-I Lr haplotypes and the cellular response was also observed, although it was only statistically significant when using the LR χ^2^ test (α = 0.046) (Figure 2C, Table 4). On the other hand, no significant correlations were observed with any of the tests used among SLA-I or SLA-II Lr haplotypes and antibody responses (Figure 2B,D, Table 4), although a trend in correlation was observed for the combination of SLA-I and SLA-II Lr haplotypes, only supported by a statistically significant χ^2^ test (χ^2^ α = 0.042, LR χ^2^ α = 0.914) with a high Cramer’s value, Cv = 0.81 (Table 4).

**Figure 3:**
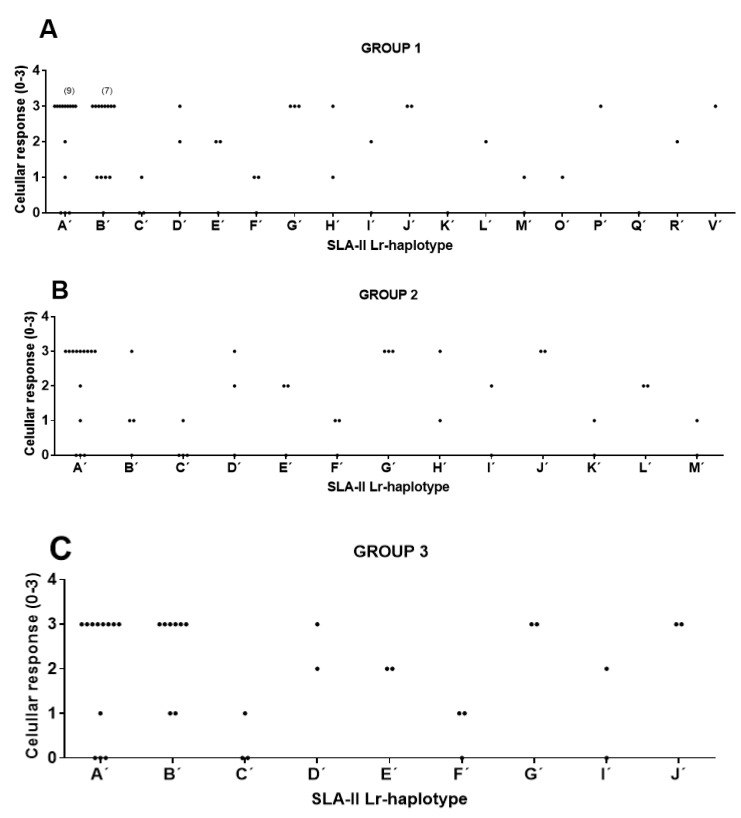
Significant correlations between SLA-II Low-resolution (Lr) haplotypes and cellular response in immunized pigs from groups 1, 2, and 3. Animal group composition as in Table S3. Cellular response values as in Table S2 and Table 1. (**A**) Group 1. Significance: χ^2^ α = 0.022 *, LR χ^2^ α = 0.034 *, Fisher’s exact α = 0.003 *, Cv = 0.66. (**B**) Group 2. Significance: χ^2^ α = 0.010 *, LR χ^2^ α = 0.014 *, Fisher’s exact α = 0.001 *, Cv = 0.67. (**C**) Group 3. Significance: χ^2^ α = 0.004 *, LR χ^2^ α = 0.008 *, Fisher’s exact α = 0.001 *, Cv = 0.67. IFN-γ secreting T cell values were categorized as: 0: <50; 1: >50 and <90; 2: >90 and <140; 3: >140.

**Figure 4:**
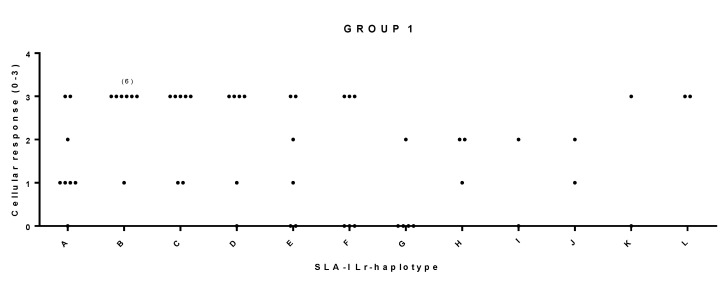
Significant correlation between SLA-I Low-resolution (Lr) haplotype and cellular response in pigs from group 1. Animal group composition as in Table S3, cellular response values as in Table S2 and Figure S1. Significance: χ^2^ α = 0.021 *, LR χ^2^ α = 0.039 *, Fisher’s exact α = 0.011 *, Cv = 0.64. IFN-γ secreting T cell values were categorized as: 0: <50; 1: >50 and <90; 2: >90 and <140; 3: >140.

When animals with haplotypes represented only once were removed from the comparison, the size of the samples allowed using the Fisher’s test, and only correlations giving positive values with χ^2^, LR χ^2^, and Fisher’s tests were considered as significant (α < 0.05) (Table 4). In this case, the three groups of compared animals corresponded to those in which the following haplotypes were represented more than once in the sample: SLA-I Lr haplotypes (group 1), SLA-II Lr haplotypes (group 2), and the third group including pigs with SLA-I and SLA-II Lr haplotype combinations represented more than once (group 3) (Table S3). Robust significant correlations between SLA-II Lr haplotypes and cellular response were observed in animals from group 1 (χ^2^ α = 0.022, LR χ^2^ α = 0.034, Fisher’s exact α = 0.003), group 2 (χ^2^ α = 0.010, LR χ^2^ α = 0.014, Fisher’s exact α = 0.001), and group 3 (χ^2^ α = 0.004, LR χ^2^ α = 0.008, Fisher’s exact α = 0.001) (Figure 3). Cramer’s values over 0.6 evidenced strong association among variables (Cv = 0.66 for group 1, Cv = 0.67 for group 2 and Cv = 0.67 for group 3) (Table 4). This result supports a clear correlation between the haplotype and the magnitude of the specific SLA-II restricted T-cells elicited by the B_2_T dendrimers in pigs. A similar correlation with SLA-I Lr haplotype and the cellular response was observed only in animals from group 1 (χ^2^ α = 0.021, LR χ^2^ α = 0.039, Fisher’s exact α = 0.011), with a Cv of 0.64 (Figure 4, Table 4). A trend of correlation supported by a single statistical significance in Fisher’s exact test was also found in pigs from group 2 (χ^2^ α = 0.097, LR χ^2^ α = 0.051, Fisher’s exact α = 0.041) (Table 4), suggesting the contribution of SLA-I restricted T-cells in vitro IFN-γ-production elicited by B_2_T dendrimers. Moreover, in group 3, a partial correlation supported by the significance of two of the three statistical tests was also observed between cellular response and the combination of the two Lr haplotypes SLA-I+SLA-II (χ^2^ α = 0.034, LR χ^2^ α = 0.068, Fisher’s exact α = 0.029, Cv = 0.71) (Table 4).

**Figure 5:**
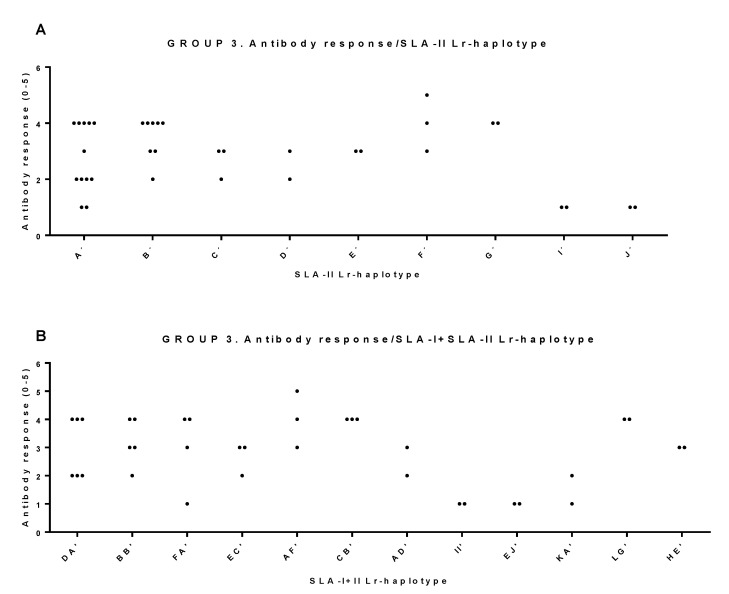
Significant correlations between SLA-II and SLA-I+SLA-II Low-resolution (Lr) haplotypes and antibody response in group 3. Animal group composition as in Table S3, antibody response values as in Table S2 and Figure S1. (**A**) Antibody response and SLA-II haplotype. Significance: χ^2^ α = 0.010 *, LR χ^2^ α = 0.019 *, Fisher’s exact α = 0.011 *, Cv = 0.65. (**B**) Antibody response and SLA-I+SLA-II haplotype. Significance: χ^2^ α = 0.030 *, LR χ^2^ α = 0.022 *, Fisher’s exact α = 0.020 *, Cv = 0.72. VNT were categorized as: 0: <0.0; 1: >0.9 and <1.5; 2: >1.5 and <1.8; 3: >1.8 and <2.4; 4: >2.4 and <3; 5: >3.

On the other hand, the only robust significant antibody response correlations were found for SLA-II Lr haplotypes (χ^2^ α = 0.010, LR χ^2^ α = 0.019, Fisher’s exact α = 0.011, Cv = 0.65), as well as for the combination of SLA-I+SLA-II Lr haplotypes (χ^2^ α = 0.030, LR χ^2^ α = 0.022, Fisher’s exact α = 0.020, Cv = 0.72) in pigs belonging to group 3. This indicates that the SLA-II Lr haplotype influences the magnitude of the antibody response either alone or in combination with SLA-I Lr haplotypes (Figure 5, Table 4). Indeed, a partial correlation endorsed by statistical significance in two of the three tests was observed for group 3 SLA-I Lr haplotypes (χ^2^ α = 0.067, LR χ^2^ α = 0.024, Fisher’s exact α = 0.044, Cv = 0.72) and also in group 1 (χ^2^ α = 0.029, LR χ^2^ α = 0.095, Fisher’s exact α = 0.012, Cv = 0.62). For group 2, a trend of correlation only supported by Fisher’s exact test significance was found (χ^2^ α = 0.123, LR χ^2^ α = 0.107, Fisher’s exact α = 0.041, with a Cv = 0.58) (Table 4).

Finally, in group 2, statistical significance only observed in χ^2^ tests, suggested a faint correlation between antibody response and SLA-II Lr haplotypes (χ^2^ α = 0.050, LR χ^2^ α = 0.264, Fisher’s exact α = 0.074, Cv = 0.62) and in the combination of SLA-I and SLA-II Lr haplotypes (χ^2^ α = 0.035, LR χ^2^ α = 0.416, Fisher’s exact α = 0.807, Cv = 0.8) (Table 4).

### 3.3. Association of SLA-I and SLA-II Haplotypes with High Immune Responses

Next, we searched for SLA Lr haplotypes associated with high immune responses. Shared SLA Lr haplotypes among animals eliciting higher antibody response (values of 5 and 4 in Table S2) and cellular response (value of 3 in Table S2) are shown in Table 3. Strong involvement in the T-cell response was mostly observed for Lr-Hp 22.0, 59.0, and 37.0 (SLA-I) and Lr-Hp 0.15b and 0.27 (SLA-II). Likewise, the Lr haplotypes associated with high antibody titers were Lr-Hp 59.0 and 22.0 (SLA-I) and Lr-Hp 0.15b and 0.27 (SLA-II).

The allele-group combinations mainly associated with low antibody responses (value of 1 in Table S2) were Lr-Hp 37.0. (SLA-I) and Lr-Hp 0.15b and 0.24 (SLA-II). Interestingly, while Lr-0.15b is one of the most frequently represented haplotypes and it also appeared in the group of high antibody responders, Lr-0.24, although less frequent, is present only in animals with low antibody responses (Table 5).

## 4. Discussion

Incorporation of epitopes efficiently recognized by both B- and T-cells, ideally with the latter being widely recognized and presented by MHC alleles frequently represented in host populations, is a crucial issue for developing efficient new FMD vaccines based on virus subunits, such as peptide vaccines.

Here, we have analyzed three SLA class I loci and three SLA class II loci to search for possible correlations between individual allele-groups and Lr haplotypes with the outcome of the antibody and the cellular responses elicited by B_2_T, which had induced high levels of neutralizing antibodies, as well as evoked significant T-cell responses, supporting the requirement of specific T-cell help for the induction of FMDV neutralizing antibodies in this species.

A cohort of 63 pigs, immunized with FMDV B_2_T dendrimers, and 10 non-immunized (control) animals, was analyzed. In these animals 23 SLA class allele-groups, and 23 SLA class II allele-groups at the three SLA loci for both SLA gene clusters were found. The found allele-groups contribute to 15 SLA-I and 16 SLA-II Lr-Hp, reflecting the presumed high level of SLA polymorphism in farmed pigs. For example, among a studied cohort of 518 European farmed pigs, 51 SLA-I and 36 SLA-II Lr-Hp have been described (S.E. Hammer, unpublished data).

The association found between the polymorphism in SLA molecules and the neutralizing antibodies and T-cell responses induced by FMD dendrimer peptide vaccines in swine is similar to that reported in cattle between polymorphism in bovine BoLA class II molecules elicited by linear FMDV peptides in cattle [2]. Nevertheless, while for linear peptides, the only significant correlation was observed between certain BoLA class II allele groups and lack of protective response in cattle, our results point to a robust significant correlation in the 63 immunized pigs (group 0) analyzed between SLA-II Lr haplotypes and the T-cell response.

Studies on the correlation of SLA polymorphisms and phenotypic traits have to face the need for a large number of individuals to be able to obtain significant results. In this scenario, the selection of the statistical analyses and the criteria for significance followed turns essential. Thus, we adopted a strict criterion to consider a correlation as statistically robust being significant when using Pearson Chi-square (χ^2^) test—which adjusts to correlation analyses using categorical values and multiple groups—, the maximum likelihood ratio chi-square (LR χ^2^) test, and when feasible, the Fisher’s exact test. In addition, Cramer’s value (Cv) has been calculated, giving robustness to the correlation study as it indicates the degree of correlation between analyzed variables.

In our complete cohort (73 pigs), the proportion of Lr haplotypes found in a single animal was 36% for SLA-I, and 40% for SLA-II, which limited the significance of the comparisons. Indeed, that was reflected in the fact that the correlation between SLA-II and the T-cell response was the only scored as significant with the tests used. When animals with haplotypes represented were only removed once from the analysis, several significant correlations were observed, entrenching the value of the analysis. Overall, these results support the contribution of SLA-II restricted T-cells to the magnitude of the specific SLA-II restricted T-cells evoked by the B_2_T dendrimers in pigs.

On the other hand, full significant correlations with B-cell antibody response were only observed in group 3 (comprising the combination of SLA-I and SLA-II Lr haplotypes represented in the cohort), with both SLA-II and SLA-II+SLA-I giving full significant correlations.

The correlation of T-cell response with SLA-I haplotype was not as evident as with SLA-II, since robust correlations were only observed when the cohort was reduced to animals with SLA-I Lr haplotypes represented more than once. These results are consistent with the kind of T-cell epitopes included in the dendrimers, as they were previously identified as swine T helper epitopes, mainly recognized by SLA class II molecules [21,23]. No robust significant correlation between antibody response and SLA-I Lr haplotypes was found in any of the groups, which is consistent with a limited implication of class I-restricted responses in B-cell activation.

The implication of single Lr haplotypes was also noticed in the magnitude of the specific responses elicited in immunized pigs. Strong involvement in the T-cell response was observed for Lr-Hp 22.0, 59.0, and 37.0 (SLA-I) and Lr-Hp 0.15b and 0.27 (SLA-II). Likewise, the allele-groups associated with high antibody titers were Lr-Hp 59.0 and 22.0 (SLA-I) and Lr-Hp 0.15b and 0.27 (SLA-II). Interestingly, one of the SLA-II Lr haplotypes, Lr-0.24, was found mostly associated with low antibody responses.

SLA-I Lr-Hp 59.0, 22.0, and 37.0 are the most frequently represented in animals with high humoral and cellular responses, but Lr-37.0 is also highly represented in animals with low antibody titers. As Lr-Hp 37.0 and 59.0 share the allele group SLA-3*05XX, the other non-shared allele groups could be responsible for these differences. So, SLA-1*08XX, and 11XX, SLA-306XX and SLA-216:02 and *12XX allele groups could be associated with higher immune responses.

The most frequent SLA-II Lr haplotype is 0.15b, represented at a high rate in animals with high immune responses, while SLA-II Lr haplotype 0.27 is only present in animals with high antibody and cellular response. Two of the allele groups belonging to this haplotype (DRB1*09XX and DQB1*09XX) could also be related to high immune responses. The third allele group (DQA*04XX), which is also present in Lr haplotype 0.21, is represented in animals with high antibody titers, but not high T-cell responses, and is mainly associated with a high humoral response.

SLA-II haplotype Lr-0.24, which was represented in only eight of the immunized pigs, was not found among the high antibody responder animals, suggesting that its presence might lead to lower antibody responses. As Lr-Hp 0.15b and 0.24 haplotypes share the same allele groups for the *DQB1*, and *DQA* loci, a possible association of DRB1*07XX allele group, only present in Lr-Hp 0.24, with a low humoral response, could be found out.

The correlations found could bear potential, as SLA-I Lr-Hp Lr-59.0 and Lr-22.0, as well as Lr-0.15b, are highly abundant Lr haplotypes in European farmed pigs [S.E. Hammer, unpublished data].

Despite part of the pigs were challenged with FMDV (experiments 2 and 3), attempts to correlate protection and SLA Lr haplotypes were not possible, due to the limited sample size. Nevertheless, the results presented here and previous results with B_2_T dendrimer peptides in which no SLA typing was available [22,26] show a high level of protection and support a clear trend between high neutralizing antibody titers and protection against FMDV challenge.

## 5. Conclusions

This study of the contribution of the polymorphism of the porcine MHC on the immunogenicity of B_2_T dendrimers FMDV displaying B- and T-cell FMDV epitopes supports the contribution of SLA-II restricted T-cells to the magnitude of the T-cell response, and slightly less significantly, to the antibody response evoked by the B_2_T dendrimers. These results are of potential value for FMD peptide vaccine design.

## Figures and Tables

**Table 1:** B_n_T_n_ dendrimeric constructions.

	B_2_T	B_2_TT	B_2_T-TB_2_
General Structure ^1^	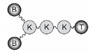	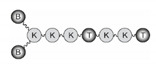	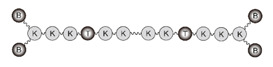
B epitope	acetyl-PVTNVRGDLQVLAQKAARTC-amide		
T-3A	AAIEFFEGMVHDSIK-amide		
T-3D	IFSKHRGDTKMSAED-amide		
Peptides	B_2_T-3A B_2_T-3D	B_2_TT-3A3D B_2_TT-3D3A	B_2_T-3A-3A-TB_2_
MW ^2^	6742.8 Da 6770.8 Da	8708.1 Da	14,247.6 Da
HPLC ^3^	6.9 min (98%) 5.1 min (98%)	6.2 min (98%) 6.0 min (97%)	7.9 min (97%)

^1^ B_n_T_n_ constructs with n B-epitope copies linked to one or two T-epitopes (T-3A or T-3D) in different dendrimeric architectures. Briefly, two C-terminally thiol-functionalized B-cell epitope branches were connected via maleimide linkages at both α- and ε-amino ends of a branched Lys core T-epitope. B_2_T-TB_2_ platform was synthesized by tail-to-tail fusion of two B_2_T maleimide subunits via orthogonal chemical ligation by copper(I)-catalyzed azide-alkyne 1,3-cycloaddition (CuAAC). ^2^ Experimental mass determined by LC/MS. ^3^ HPLC retention time (Phenomenex Luna C_18_ column, 4.6 × 50 mm, 3 µm) eluted with a 20–60% linear gradient of solvent B (0.036% TFA in MeCN) into solvent A (0.045% TFA in H_2_O) over 15 min. In parenthesis, HPLC homogeneity of purified material.

**Table 2:** Pigs immunized with dendrimer peptides analyzed in this study.

Experiment ^1^	Peptide Vaccine	Amount (mg)	Boost	Animal Number
1 [26,28]	B_2_T-3A	2	Yes	Sw01–04
	B_2_T-TB_2_	2	Yes	Sw05–08
	B_2_T-TB_2_	0.5	Yes	Sw09–12
	PBS	-	-	Sw13, 14
2 [24]	B_4_T	2	Yes	Sw15, 16, 21, 23, 24, 30
	B_2_T-3A	2	Yes	Sw17–19, 26, 27, 29
	B_2_T-THIO	2	Yes	Sw20, 22, 25, 28, 31
3 [25]	B_2_T-3A	2	No	Sw32–36
	B_2_T-3A	0.5	No	Sw37–41
	PBS	-	-	Sw42, 43
4 [27]	B_2_T-3A	2	Yes	Sw44–47
	B_2_T-3D	2	Yes	Sw48–51
	PBS	-	-	Sw52, 53
5 [P. De León, manuscript in preparation]	B_2_T-3A	2	Yes	Sw54–57
	B_2_T-3D	2	Yes	Sw58–61
	B_2_TT-3A3D	2	Yes	Sw62–65
	B_2_TT-3D3A	2	Yes	Sw66–69
	B_2_	2	Yes	Sw70, 71
	PBS	-	-	Sw72, 73

^1^ The pigs included in this study belonged to five independent experiments, for which references are provided.

**Table 3:** SLA-I and SLA-II Low-resolution Haplotype notation and occurrence.

	Lr-Hp SLA-I ^1^	Number ^2^		Lr-Hp SLA-II ^1^	Number ^2^
A	59.0/1.0	8	A’	0.15b/0.15b	14
B	22.0/59.0	7	B’	0.15b/0.27	12
C	37.0/59.0	7	C’	0.1/0.15b	4
D	22.0/37.0	6	D’	0.27/0.4	3
E	1.0/37.0	6	E’	0.15b/0.24	3
F	1.0/22.0	6	F’	0.1/0.27	3
G	59.0/4.0	5	G’	0.15b/0.21	3
H	29.0/37.0	3	H’	0.15b/0.25	2
I	57.0/37.0	2	I’	0.24/0.33	2
J	6.0/4.0	2	J’	0.24/0.24	2
K	37.0/37.0	2	K’	0.23/0.5	2
L	7.0/22.0	2	L’	0.15b/0.23	2
M	1.0/6.0	1	M’	0.27/0.23	2
N	6.0/46.0	1	N’	0.15b/0.5	1
O	33.0/28.0	1	O’	LWLR-1/0.27	1
P	6.0/24.0	1	P’	0.24/0.27	1
Q	1.0/4.0	1	Q’	0.27/0.5	1
R	22.0/24.0	1	R’	0.23/0.22	1
S	22.0/11.0	1	S’	0.1/0.22	1
			T’	0.27/0.22	1
			U’	0.23/0.11	1
			V’	0.13/0.27	1

^1^ Capital letters were assigned to the different SLA-I and SLA-II (letter ’) allele-group combinations (Low-resolution Haplotypes, Lr-Hp). ^2^ Number of pigs showing this haplotype.

**Table 4:** Data from statistical tests resulting from analysis of the correlation between antibody/cellular response and Lr-haplotypes in groups 0, 1, 2, and 3.

SLA Lr Haplotype			Cellular ^1^			Antibody ^1^		
			SLA-I	SLA-II	SLA-I/II	SLA-I	SLA-II	SLA-I/II
Group 0	χ^2^		0.053	**0.015**	0.099	0.065	0.102	**0.042**
	LR χ^2^		**0.046**	**0.038**	0.265	0.251	0.634	0.914
	Cv		0.59	0.66	0.81	0.58	0.61	0.81
Group 1	χ^2^		**0.021**	**0.022**	0.061	**0.029**	0.082	0.055
	LR χ^2^		**0.039**	**0.034**	0.168	0.095	0.358	0.502
	Fisher’s	exact	**0.011**	**0.003**	0.81	0.012	0.125	0.806
	Cv		0.64	0.66	0.81	0.62	0.61	0.8
Group 2	χ^2^		0.097	**0.01**	0.054	0.123	**0.05**	**0.035**
	LR χ^2^		0.051	**0.014**	0.111	0.107	0.264	0.416
	Fisher’s	exact	**0.041**	**0.001**	0.8	**0.041**	0.074	0.807
	Cv		0.6	0.67	0.8	0.58	0.62	0.8
Group 3	χ^2^		0.077	**0.004**	**0.034**	0.067	**0.01**	**0.03**
	LR χ^2^		0.128	**0.008**	0.068	**0.024**	**0.019**	**0.022**
	Fisher’s	exact	0.148	**0.001**	**0.029**	**0.044**	0.011	0.02
	Cv		0.64	0.67	0.71	0.72	0.65	0.72

^1^ Bold numbers indicate that the test is statistically significant (a < 0.05).

**Table 5:** Shared allele-groups and Low-resolution Haplotypes among the T-cell high responding animals and antibody high and low responder animals.

SLA-I, HIGH T-Cell Response ^1^					SLA-I, HIGH Antibody Response ^2^				
SLA-1	SLA-3	SLA-2	Lr-Hp ^4^	Haplotype ^5^	SLA-1	SLA-3	SLA-2	Lr-Hp ^4^	Haplotype ^5^
08XX	07XX	05XX	7.0 (2)	L	01XX	01XX	01XX	1.0 (4)	A, F
01XX	01XX	01XX	1.0 (7)	A, E, F	04XX	04XX	04XX	4.0 (5)	G
*07XX*	*05XX*	*09XX*	*37.0 (12)*	*C, D, E, K*	*07XX*	*05XX*	*09XX*	*37.0 (7)*	*C, D*
*11XX*	*05XX*	*16:02*	*59.0 (13)*	*A, B, C*	*08XX*	*06XX*	*12XX*	*22.0 (12)*	*B, D, F, L, R, S*
*08XX*	*06XX*	*12XX*	*22.0 (15)*	*B, D, F, L*	*11XX*	*05XX*	*16:02*	*59.0 (12)*	*A, B, C, G*
SLA-I, LOW Antibody Response ^3^					SLA-II, HIGH T-Cell Response ^1^				
SLA-1	SLA-3	SLA-2	Lr-Hp ^4^	Haplotype ^5^	DRB1	DQB1	DQA	Lr-Hp ^4^	Haplotype ^5^
02XX	01XX	11XX	57.0 (2)	I	02XX	04XX	02XX	0.4 (1)	D’
11XX	05XX	16:02	59.0 (3)	B, C	04XX	03XX	02XX	0.13 (1)	V’
08XX	06XX	12XX	22.0 (4)	B, F	01XX	05XX	04XX	0.21 (3)	G’
01XX	01XX	01XX	1.0 (5)	E, F	07XX	02XX	02XX	0.24 (3)	J’, P’
*07XX*	*05XX*	*09XX*	*37.0 (8)*	*C, E, I, K*	*09XX*	*09XX*	*04XX*	*0.27 (10)*	*B’, D’, P’, V’*
					*04XX*	*02XX*	*02XX*	*0.15b (20)*	*A’, B’, G’, H’*
SLA-II, HIGH Antibody Response ^2^					SLA-II, LOW Antibody Response ^3^				
DRB1	DQB1	DQA	Lr-Hp ^4^	Haplotype ^5^	DRB1	DQB1	DQA	Lr-Hp ^4^	Haplotype ^5^
01XX	05XX	04XX	0.21 (3)	G’	*07XX*	*02XX*	*02XX*	*0.24 (8)*	*J’, P’*
10XX	06XX	01XX	0.23 (5)	K’, L’, M’, U’	*04XX*	*02XX*	*02XX*	*O.15b (9)*	*A’, B’, E’, H’*
*09XX*	*09XX*	*04XX*	*0.27 (11)*	*B’, D’, P’, V’*					
*04XX*	*02XX*	*02XX*	*0.15b (20)*	*A’, B’, G’, H’*					

^1^ High response: IFN-γ secreting T cell values of category 3 in Table S2. ^2^ High response: VNT categories 5 and 4 in Table S2. Only those allele-groups represented more than 3 times in the sample are indicated. ^3^ Low response: VNT categories 1 in Table S2. Only those allele-groups represented more than 2 times in the sample are indicated. ^4^ In brackets, number of animals with the indicated haplotype within each group (^1, 2^ or ^3^). ^5^ As in Table 3. In italics, allele-group combinations (Low-resolution Haplotypes, Lr-Hp) represented by a greater number of animals within each group (^1, 2^ or ^3^).

## Supplementary Materials

The following are available online at https://www.mdpi.com/2076-393X/8/3/513/s1. Figure S1: Antibody and cellular responses of 73 pigs to dendrimeric peptides. Table S1: SLA-I and SLA-II allele-groups and Low-resolution Haplotypes in the cohort of 73 studied pigs; Table S2: SLA-I and SLA-II Low-resolution Haplotypes of 73 pigs and their antibody and cellular responses to dendrimeric peptides; Table S3: Groups of pigs with Low-resolution Haplotypes (Lr-Hp) represented more than once.

## References

[1] Blanco E., Andreu D., Sobrino F., Sobrino F., Domingo E. (2017). Advantages, challenges and future of peptide vacines. Foot-and-Mouth Disease Virus. Current Research and Emerging Trends.

[2] Garcia-Briones M.M., Russell G.C., Oliver R.A., Tami C., Taboga O., Carrillo E., Palma E.L., Sobrino F., Glass E.J. (2000). Association of bovine DRB3 alleles with immune response to FMDV peptides and protection against viral challenge. Vaccine.

[3] Kulski J.K., Shiina T., Dijkstra J.M. (2019). Genomic Diversity of the Major Histocompatibility Complex in Health and Disease. Cells.

[4] Grubman M.J., Baxt B. (2004). Foot-and-mouth disease. Clin. Microbiol. Rev..

[5] Robinson L., Knight-Jones T.J., Charleston B., Rodriguez L.L., Gay C.G., Sumption K.J., Vosloo W. (2016). Global Foot-and-Mouth Disease Research Update and Gap Analysis: 7—Pathogenesis and Molecular Biology. Transbound. Emerg. Dis..

[6] Knight-Jones T.J., Robinson L., Charleston B., Rodriguez L.L., Gay C.G., Sumption K.J., Vosloo W. (2016). Global Foot-and-Mouth Disease Research Update and Gap Analysis: 1—Overview of Global Status and Research Needs. Transbound. Emerg. Dis..

[7] Cao Y., Lu Z., Liu Z. (2016). Foot-and-mouth disease vaccines: Progress and problems. Expert Rev. Vaccines.

[8] Kleid D.G., Yansura D., Small B., Dowbenko D., Moore D.M., Grubman M.J., McKercher P.D., Morgan D.O., Robertson B.H., Bachrach H.L. (1981). Cloned viral protein vaccine for foot-and-mouth disease: Responses in cattle and swine. Science.

[9] Bittle J.L., Houghten R.A., Alexander H., Shinnick T.M., Sutcliffe J.G., Lerner R.A., Rowlands D.J., Brown F. (1982). Protection against foot-and-mouth disease by immunization with a chemically synthesized peptide predicted from the viral nucleotide sequence. Nature.

[10] Strohmaier K., Franze R., Adam K.H. (1982). Location and characterization of the antigenic portion of the FMDV immunizing protein. J. Gen. Virol..

[11] DiMarchi R., Brooke G., Gale C., Cracknell V., Doel T., Mowat N. (1986). Protection of cattle against foot-and-mouth disease by a synthetic peptide. Science.

[12] Doel T.R., Gale C., Brooke G., DiMarchi R. (1988). Immunization against foot-and-mouth disease with synthetic peptides representing the C-terminal region of VP1. J. Gen. Virol..

[13] Collen T., Dimarchi R., Doel T.R. (1991). A T cell epitope in VP1 of foot-and-mouth disease virus is immunodominant for vaccinated cattle. J. Immunol..

[14] Firbas C., Jilma B., Tauber E., Buerger V., Jelovcan S., Lingnau K., Buschle M., Frisch J., Klade C.S. (2006). Immunogenicity and safety of a novel therapeutic hepatitis C virus (HCV) peptide vaccine: A randomized, placebo controlled trial for dose optimization in 128 healthy subjects. Vaccine.

[15] Kunwar P., Hawkins N., Dinges W.L., Liu Y., Gabriel E.E., Swan D.A., Stevens C.E., Maenza J., Collier A.C., Mullins J.I. (2013). Superior control of HIV-1 replication by CD8+ T cells targeting conserved epitopes: Implications for HIV vaccine design. PLoS ONE.

[16] Taboga O., Tami C., Carrillo E., Nunez J.I., Rodriguez A., Saiz J.C., Blanco E., Valero M.L., Roig X., Camarero J.A. (1997). A large-scale evaluation of peptide vaccines against foot-and-mouth disease: Lack of solid protection in cattle and isolation of escape mutants. J. Virol..

[17] Gao F.S., Zhai X.X., Jiang P., Zhang Q., Gao H., Li Z.B., Han Y., Yang J., Zhang Z.H. (2018). Identification of two novel foot-and-mouth disease virus cytotoxic T lymphocyte epitopes that can bind six SLA-I proteins. Gene.

[18] Pedersen L.E., Patch J.R., Kenney M., Glabman R.A., Nielsen M., Jungersen G., Buus S., Golde W.T. (2016). Expanding specificity of class I restricted CD8(+) T cells for viral epitopes following multiple inoculations of swine with a human adenovirus vectored foot-and-mouth disease virus (FMDV) vaccine. Vet. Immunol. Immunopathol..

[19] Ning S., Wang Z.B., Qi P., Xiao J., Wang X.J. (2020). Crystallization of SLA-2*04:02:02 complexed with a CTL epitope derived from FMDV. Res. Vet. Sci..

[20] Tam J.P. (1988). Synthetic peptide vaccine design: Synthesis and properties of a high-density multiple antigenic peptide system. Proc. Natl. Acad. Sci. USA.

[21] Blanco E., Garcia-Briones M., Sanz-Parra A., Gomes P., De Oliveira E., Valero M.L., Andreu D., Ley V., Sobrino F. (2001). Identification of T-cell epitopes in nonstructural proteins of foot-and-mouth disease virus. J. Virol..

[22] Cubillos C., de la Torre B.G., Jakab A., Clementi G., Borras E., Barcena J., Andreu D., Sobrino F., Blanco E. (2008). Enhanced mucosal immunoglobulin A response and solid protection against foot-and-mouth disease virus challenge induced by a novel dendrimeric peptide. J. Virol..

[23] Garcia-Briones M.M., Blanco E., Chiva C., Andreu D., Ley V., Sobrino F. (2004). Immunogenicity and T cell recognition in swine of foot-and-mouth disease virus polymerase 3D. Virology.

[24] Blanco E., Guerra B., de la Torre B.G., Defaus S., Dekker A., Andreu D., Sobrino F. (2016). Full protection of swine against foot-and-mouth disease by a bivalent B-cell epitope dendrimer peptide. Antivir. Res..

[25] Cañas-Arranz R., Forner M., Defaus S., de Leon P., Bustos M.J., Torres E., Sobrino F., Andreu D., Blanco E. (2020). A Single Dose of Dendrimer B_2_T Peptide Vaccine Partially Protects Pigs against Foot-and-Mouth Disease Virus Infection. Vaccines.

[26] Cañas-Arranz R., Forner M., Defaus S., Rodriguez-Pulido M., de Leon P., Torres E., Bustos M.J., Borrego B., Saiz M., Blanco E. (2020). A bivalent B-cell epitope dendrimer peptide can confer long-lasting immunity in swine against foot-and-mouth disease. Transbound. Emerg. Dis..

[27] Cañas-Arranz R., de León P., Forner M., Defaus S., Bustos M.J., Torres E., Andreu D., Blanco E., Sobrino F. (2020). Immunogenicity of a dendrimer B_2_T peptide harboring a T-cell epitope from FMDV non-structural protein 3D. Front. Vet. Sci..

[28] Defaus S., Forner R., Cañas-Arranz R., de León P., Bustos M.J., Rodríguez-Pulido M., Blanco E., Sobrino F., Andreu D. (2020). Designing functionally versatile, highly immunogenic peptide-based multiepitopic vaccines against foot-and-mouth disease virus. Vaccines.

[29] Hammer S.E., Ho C.S., Ando A., Rogel-Gaillard C., Charles M., Tector M., Tector A.J., Lunney J.K. (2020). Importance of the Major Histocompatibility Complex (Swine Leukocyte Antigen) in Swine Health and Biomedical Research. Annu. Rev. Anim. Biosci..

[30] Lunney J.K., Ho C.S., Wysocki M., Smith D.M. (2009). Molecular genetics of the swine major histocompatibility complex, the SLA complex. Dev. Comp. Immunol..

[31] Monso M., de la Torre B.G., Blanco E., Moreno N., Andreu D. (2013). Influence of conjugation chemistry and B epitope orientation on the immune response of branched peptide antigens. Bioconjug. Chem..

[32] Gimsa U., Ho C.S., Hammer S.E. (2017). Preferred SLA class I/class II haplotype combinations in German Landrace pigs. Immunogenetics.

[33] Ho C.S., Lunney J.K., Franzo-Romain M.H., Martens G.W., Lee Y.J., Lee J.H., Wysocki M., Rowland R.R., Smith D.M. (2009). Molecular characterization of swine leucocyte antigen class I genes in outbred pig populations. Anim. Genet..

[34] Ho C.S., Lunney J.K., Ando A., Rogel-Gaillard C., Lee J.H., Schook L.B., Smith D.M. (2009). Nomenclature for factors of the SLA system, update 2008. Tissue Antigens.

[35] Miller R., Siegmund D. (1982). Maximally selected chi square statistics. Biometrics.

[36] McHugh M.L. (2013). The chi-square test of independence. Biochem. Med..

