# Supplementary material for: Association of Porcine Swine Leukocyte Antigen (SLA) Haplotypes with B- and T-Cell Immune Response to Foot-and-Mouth Disease Virus (FMDV) Peptides
Source: Vaccines (Basel). 2020 Sep 8;8(3):513. doi: 10.3390/vaccines8030513 (PMC7563261; doi:10.3390/vaccines8030513)
Supplement: Supplementary file 1 [file vaccines-08-00513-s001.pdf]

vaccines
MDPI

Supplementary Material

Association of porcine Swine Leukocyte Antigen (SLA) haplotypes with B- and T-cell immune response to foot-and-mouth disease virus (FMDV) peptides

Patricia de León [1,a], Rodrigo Cañas-Arranz [1,a], Yago Saez [2], Mar Forner [3], Sira Defaus [3], Dolores Cuadra [4], María J. Bustos [1], Elisa Torres [1], David Andreu [3], Esther Blanco [5], Francisco Sobrino [1,*] and Sabine E. Hammer [6,*]

[1] Centro de Biología Molecular "Severo Ochoa" (CSIC-UAM), 28049 Madrid, Spain; pdeleon@cbm.csic.es (P.L.); rcannas@cbm.csic.es (R.C-A.); mjbustos@cbm.csic.es (M.B.); elisa.torres@inia.es (E.T.); fsobrino@cbm.csic.es (F.S.)
[2] Computer Science Department, Universidad Carlos III of Madrid, Leganés, Madrid, 28911, Spain; yago.saez@uc3m.es (Y.S.)
[3] Departament de Ciències Experimentals i de la Salut, Universitat Pompeu Fabra, 08003 Barcelona, Spain; mar.forner@upf.edu (M.F.); sira.defaus@upf.edu (S.D.); david.andreu@upf.edu (D.A.)
[4] Computer Science Department, Universidad Rey Juan Carlos, Móstoles 28933, Spain; dolores.cuadra@urjc.es (D.C.)
[5] Centro de Investigación en Sanidad Animal (CISA-INIA), Valdeolmos, 28130 Madrid, Spain; blanco@inia.es (E.B.)
[6] Institute of Immunology, Department of Pathobiology, University of Veterinary Medicine Vienna, A-1210 Vienna, Austria; sabine.hammer@phylo-dat.net (S.E.H.)
* Correspondence: fsobrino@cbm.csic.es; Tel.: +34-911964493 (F.S.); sabine.hammer@phylo-dat.net; Tel.: +43-1-25077-2754 (S.E.H.)
[a] Equally contributing

Keywords: swine; foot-and-mouth disease virus; dendrimer peptide; swine leukocyte antigen; SLA polymorphism; adaptive immunity; vaccine; protection

Short Title: FMDV-MHC correlations in pigs.

Table S1. SLA-I and SLA-II allele-groups and Low-resolution Haplotypes in the cohort of 73 studied pigs.

SLA class I (SLA-I)				
**SLA-1**	**SLA-3**	**SLA-2**	**Lr-Hp[1]**	**Animal number**
01XX	01XX	01XX	1.0	Sw01,04,05,07,08,09,11,12,15,23,24,27,29,33,35,44,45,46,47,48,50,51,52,72
04XX	04XX	04XX	4.0	Sw18,20,21,22,25,26,27,30
08XX	06XX	05XX	6.0	Sw15,16,19,20,26
08XX	07XX	05XX	7.0	Sw54,59
01XX,09XX	07XX	05XX	11.0	Sw31
08XX	06XX	12XX	22.0	Sw03,04,12,13,14,28,31,32,34,38,39,40,41,42,43,46,47,50,51,54,55,56,57,58,59,63,64,73
Blank[2]	04XX	06XX	24.0	Sw19,28
09XX,15XX	07XX	05XX	28.0	Sw17
Blank[2]	05XX	09XX	29.0	Sw60,68,69,70,71
Blank[2]	05XX	06XX	33.0	Sw17
07XX	05XX	09XX	37.0	Sw02,05,06,07,08,10,11,13,32,33,34,36,37,38,40,48,49,5556,60,61,62,65,66,67,68,69,70,71,72,73
12XX	04XX	06XX	46.0	Sw16
15XX	04XX	11:04	55.0	Sw14,53
02XX	01XX	11XX	57.0	Sw02,10
11XX	05XX	16:02	59.0	Sw01,03,06,09,18,21,22,23,24,25,29,30,35,39,41,42,43,44,45,49,52,53,57,58,61,62,63,64,65,66,67

SLA class II (SLA-II)				
**DRB1**	**DQB1**	**DQA**	**Lr-Hp[1]**	**Animal number**
01XX	01XX	01XX	0.1	Sw05,08,15,23,24,27,33,35
02XX	02XX	02XX	0.2	Sw72
02XX	04XX	02XX	0.4	Sw01,09,18
05XX	02XX	02XX	0.5	Sw14,16,17,21,22
05XX	08XX	01XX	0.6	Sw53
09XX	04XX	03XX	0.11	Sw31
04XX	03XX	02XX	0.13	Sw61
04XX	02XX	02XX	0.15b	Sw03,04,05,08,12,13,14,15,16,19,20,26,32,3334,36,37,38,39,40,41,42,43,45,46,47,48,49,5051,54,55,56,57,58,59,60,62,63,64,65,66,67,6869,70,71,72,73
01XX	05XX	04XX	0.21	Sw54,59,64
06XX	02XX	02XX	0.22	Sw25,27,28
10XX	06XX	01XX	0.23	Sw17,19,20,21,25,29,30,31
07XX	02XX	02XX	0.24	Sw02,06,07,10,11,12,68,69
13XX	09XX	04XX	0.25	Sw60,66,71
09XX	09XX	04XX	0.27	Sw01,04,06,09,13,18,22,23,24,26,28,29,30,3539,4142,43,44,45,49,52,53,57,58,61,63,65,67
11XX	02XX	02XX	0.33	Sw02,10
Blank[2]	02XX	02XX	LWLR-1	Sw44,52

[1] Low-resolution Haplotype. [2] Untyped SLA class I or class II locus.

Table S2. SLA-I and SLA-II Low-resolution Haplotypes of 73 pigs and their antibody and cellular responses to dendrimeric peptides.

Vaccine	Animal number	Low-resolution Haplotype		Antibody response		T-cell response	
		SLA-I[1]	SLA-II[1]	Antibody (0-5)[2]	Antibody (log_{10})	IFN-γ (0-3)[3]	IFN-γ spots/ 10^6 PBMCs
B_2T-3A (n=28)	Sw01	59.0/1.0	0.27/0.4	2	1.7	3	183.2
	Sw02	57.0/37.0	0.24/0.33	1	0.9	2	103.2
	Sw03	22.0/59.0	0.15b/0.15b	1	1.0	3	142.7
	Sw04	1.0/22.0	0.15b/0.27	1	1.2	0	36.3
	Sw17	33.0/28.0	0.23/0.5	3	2.3	1	72.0
	Sw18	59.0/4.0	0.27/0.4	4	2.6	0	41.3
	Sw19	6.0/24.0	0.15b/0.23	5	3.0	2	102.7
	Sw26	6.0/4.0	0.15b/0.27	4	2.4	1	86.7
	Sw27	1.0/4.0	0.1/0.22	3	2.3	2	120.0
	Sw29	59.0/1.0	0.27/0.23	3	2.1	1	69.3
	Sw32	22.0/37.0	0.15b/0.15b	2	1.7	1	55.3
	Sw33	1.0/37.0	0.1/0.15b	3	2.3	0	17.0
	Sw34	22.0/37.0	0.15b/0.15b	2	1.6	3	207.7
	Sw35	59.0/1.0	0.1/0.27	3	2.3	1	67.7
	Sw36	37.0/37.0	0.15b/0.15b	2	1.6	0	8.3
	Sw37	37.0/37.0	0.15b/0.15b	1	1.3	3	174.0
	Sw38	22.0/37.0	0.15b/0.15b	2	1.5	0	2.7
	Sw39	22.0/59.0	0.15b/0.27	3	2.3	1	83.0
	Sw40	22.0/37.0	0.15b/0.15b	4	2.6	3	274.0
	Sw41	22.0/59.0	0.15b/0.27	3	2.0	3	273.3
	Sw44	59.0/1.0	LWLR-1/0.27	3	2.3	1	65.0
	Sw45	59.0/1.0	0.15b/0.27	3	2.3	3	258.3
	Sw46	1.0/22.0	0.15b/0.15b	4	2.5	3	344.7
	Sw47	1.0/22.0	0.15b/0.15b	1	0.9	0	13.3
	Sw54	7.0/22.0	0.15b/0.21	4	2.5	3	272.3
	Sw55	22.0/37.0	0.15b/0.15b	4	2.9	3	204.3
	Sw56	22.0/37.0	0.15b/0.15b	4	2.9	3	241.8
	Sw57	22.0/59.0	0.15b/0.27	2	1.6	3	389.8
B_2T-TB_2 (n=8)	Sw05	1.0/37.0	0.1/0.15b	2	1.6	0	35.7
	Sw06	37.0/59.0	0.24/0.27	1	1.3	3	210.8
	Sw07	1.0/37.0	0.24/0.24	1	1.3	3	170.3
	Sw08	1.0/37.0	0.1/0.15b	3	1.8	1	66.8
	Sw09	59.0/1.0	0.27/0.4	3	2.0	2	106.5
	Sw10	57.0/37.0	0.24/0.33	1	1.3	0	33.3
	Sw11	1.0/37.0	0.24/0.24	1	1.2	3	154.3
	Sw12	1.0/22.0	0.15b/0.24	1	1.2	0	14.5
B_2T-3D (n=8)	Sw48	1.0/37.0	0.15b/0.15b	3	2.1	2	131.7
	Sw49	37.0/59.0	0.15b/0.27	2	1.7	1	76.3
	Sw50	1.0/22.0	0.15b/0.15b	3	2.1	3	500.0
	Sw51	1.0/22.0	0.15b/0.15b	4	2.4	3	371.3
	Sw58	22.0/59.0	0.15b/0.27	4	2.8	3	298.3
	Sw59	7.0/22.0	0.15b/0.21	4	2.6	3	183.8
	Sw60	29.0/37.0	0.15b/0.25	3	2.0	1	68.3
	Sw61	37.0/59.0	0.13/0.27	4	2.7	3	398.8
B_4T (n=6)	Sw15	1.0/6.0	0.1/0.15b	2	1.7	0	26.7
	Sw16	6.0/46.0	0.15b/0.5	4	2.9	0	37.3
	Sw21	59.0/4.0	0.23/0.5	4	2.7	0	37.3
	Sw23	59.0/1.0	0.1/0.27	4	2.9	1	58.7
	Sw24	59.0/1.0	0.1/0.27	5	3.0	0	49.3

	Sw30	59.0/4.0	0.27/0.23	4	2.9	0	28.0
**B_2T-THIO (n=5)**	Sw20	6.0/4.0	0.15b/0.23	2	1.7	2	100.0
	Sw22	59.0/4.0	0.27/0.5	3	2.1	0	38.7
	Sw25	59.0/4.0	0.23/0.22	5	3.0	2	94.7
	Sw28	22.0/24.0	0.27/0.22	4	2.7	2	125.3
	Sw31	22.0/11.0	0.23/0.11	4	2.4	1	78.7
**B_2T-3A3D (n=4)**	Sw62	37.0/59.0	0.15b/0.27	4	2.5	1	75.8
	Sw63	22.0/59.0	0.15b/0.27	4	2.6	3	334.8
	Sw64	22.0/59.0	0.15b/0.21	5	3.1	3	173.3
	Sw65	37.0/59.0	0.15b/0.27	4	2.8	3	274.8
**B_2T-3D3A (n=4)**	Sw66	37.0/59.0	0.15b/0.25	1	1.0	3	197.3
	Sw67	37.0/59.0	0.15b/0.27	4	2.4	3	235.8
	Sw68	29.0/37.0	0.15b/0.24	3	2.1	2	113.3
	Sw69	29.0/37.0	0.15b/0.24	3	2.3	2	114.3
**B_2 (n=2)**	Sw70	29.0/37.0	0.15b/0.25	0	0.6	0	7.8
	Sw71	29.0/37.0	0.15b/0.25	0	0.6	0	13.3
**PBS (Control) (n=8)**	Sw13	22.0/37.0	0.15b/0.27	0	0.3	0	0.0
	Sw14	22.0/55.0	0.15b/0.5	0	0.3	0	0.0
	Sw42	22.0/59.0	0.15b/0.27	0	0.3	0	0.0
	Sw43	22.0/59.0	0.15b/0.27	0	0.5	0	0.0
	Sw52	59.0/1.0	LWLR-1/0.27	0	0.6	0	0.0
	Sw53	55.0/59.0	0.6/0.27	0	0.6	0	0.0
	Sw72	1.0/37.0	0.2/0.15b	0	0.6	0	0.0
	Sw73	22.0/37.0	0.15b/0.15b	0	0.6	0	0.0

[1] The corresponding SLA-I and SLA-II Low-resolution Haplotypes found in each animal are indicated. [2] VNT were categorized as **0**: <0.9; **1**: >0.9 and <1.5; **2**: >1.5 and <1.8; **3**: >1.8 and <2.4; **4**: >2.4 and <3; **5**: >3. [3] IFN-γ secreting T cell values were categorized as: **0**: <50; **1:** >50 and <90; **2:** >90 and <140; **3:** >140.

Table S3. Groups of pigs with Low-resolution Haplotypes (Lr-Hp) represented more than once.

Group 1			
**SLA-I**	**Lr-Hp**	**n**	**Animal number**
**A**	59.0/1.0	8	Sw01,09,23,24,29,35,44,45
**B**	22.0/59.0	7	Sw03,39,41,57,58,63,64
**C**	37.0/59.0	7	Sw06,49,61,62,65,66,67
**D**	22.0/37.0	6	Sw32,34,38,40,55,56
**E**	1.0/37.0	6	Sw05,07,08,11,33,48
**F**	1.0/22.0	6	Sw04,12,46,47,50,51
**G**	59.0/4.0	5	Sw18,21,22,25,30
**H**	29.0/37.0	3	Sw60,68,69
**I**	57.0/37.0	2	Sw02,10
**J**	6.0/4.0	2	Sw20,26
**K**	37.0/37.0	2	Sw36,37
**L**	7.0/22.0	2	Sw54,59

Group 2			
**SLA-II**	**Lr-Hp**	**n**	**Animal number**
**A'**	0.15b/0.15b	14	Sw03,32,34,36,37,38,40,46,47,48,50,51,55,56
**B'**	0.15b/0.27	12	Sw04,26,39,41,45,49,57,58,62,63,65,67
**C'**	0.1/0.15b	4	Sw05,08,15,33
**D'**	0.27/0.4	3	Sw01,09,18
**E'**	0.15b/0.24	3	Sw12,68,69
**F'**	0.1/0.27	3	Sw23,24,35
**G'**	0.15b/0.21	3	Sw54,59,64
**H'**	0.15b/0.25	2	Sw60,66
**I'**	0.24/0.33	2	Sw02,10
**J'**	0.24/0.24	2	Sw07,11
**K'**	0.23/0.5	2	Sw17,21
**L'**	0.15b/0.23	2	Sw19,20
**M'**	0.27/0.23	2	Sw29,30

Group 3			
**SLAI/II**	**Lr-Hp**	**n**	**Animal number**
**D A'**	22.0/37.0 0.15b/0.15b	6	Sw32,34,38,40,55,56
**B B'**	22.0/59.0 0.15b/0.27	5	Sw39,41,57,58,63
**F A'**	1.0/22.0 0.15b/0.15b	4	Sw46,47,50,51
**E C'**	1.0/37.0 0.1/0.15b	3	Sw05,08,33
**A F'**	59.0/1.0 0.1/0.27	3	Sw23,24,35
**C B'**	37.0/59.0 0.15b/0.27	3	Sw62,65,67
**A D'**	59.0/1.0 0.27/0.4	2	Sw01,09
**I I'**	57.0/37.0 0.24/0.33	2	Sw02,10
**E J'**	1.0/37.0 0.24/0.24	2	Sw07,11
**K A'**	37.0/37.0 0.15b/0.15b	2	Sw36,37
**L G'**	7.0/22.0 0.15b/0.21	2	Sw54,59
**H E'**	29.0/37.0 0.15b/0.24	2	Sw68,69

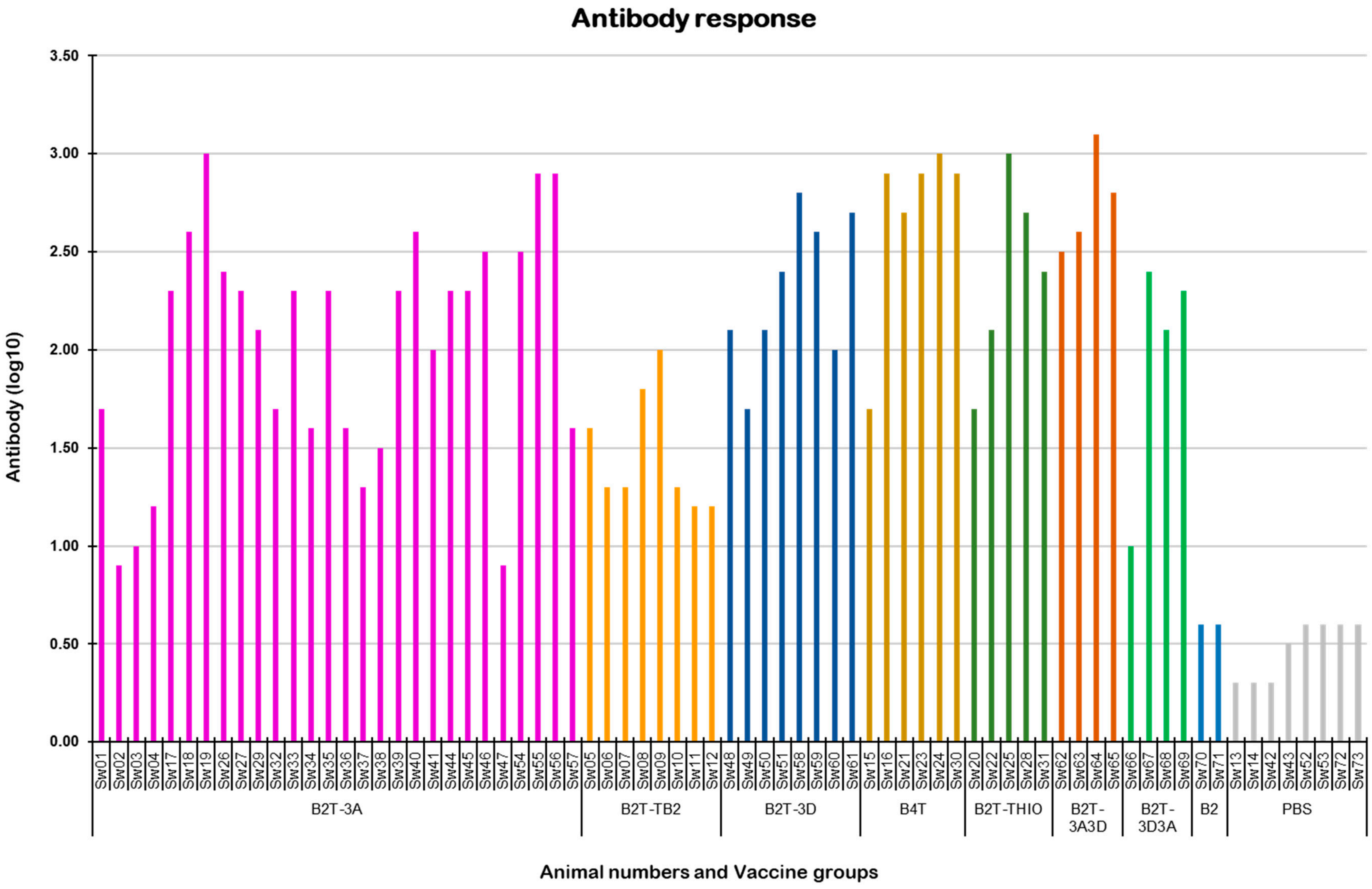
Antibody response
Antibody (log10)
3.50
3.00
2.50
2.00
1.50
1.00
0.50
0.00
Sw01
Sw02
Sw03
Sw04
Sw17
Sw18
Sw19
Sw26
Sw27
Sw29
Sw32
Sw33
Sw34
Sw35
Sw36
Sw37
Sw38
Sw39
Sw40
Sw41
Sw44
Sw45
Sw46
Sw47
Sw54
Sw55
Sw56
Sw57
Sw05
Sw06
Sw07
Sw08
Sw09
Sw10
Sw11
Sw12
Sw48
Sw49
Sw50
Sw51
Sw58
Sw59
Sw60
Sw61
Sw15
Sw16
Sw21
Sw23
Sw24
Sw30
Sw20
Sw22
Sw25
Sw28
Sw31
Sw62
Sw63
Sw64
Sw65
Sw66
Sw67
Sw68
Sw69
Sw70
Sw71
Sw13
Sw14
Sw42
Sw43
Sw52
Sw53
Sw72
Sw73
B2T-3A
B2T-TB2
B2T-3D
B4T
B2T-THIO
B2T-3A3D
B2T-3D3A
B2
PBS
Animal numbers and Vaccine groups

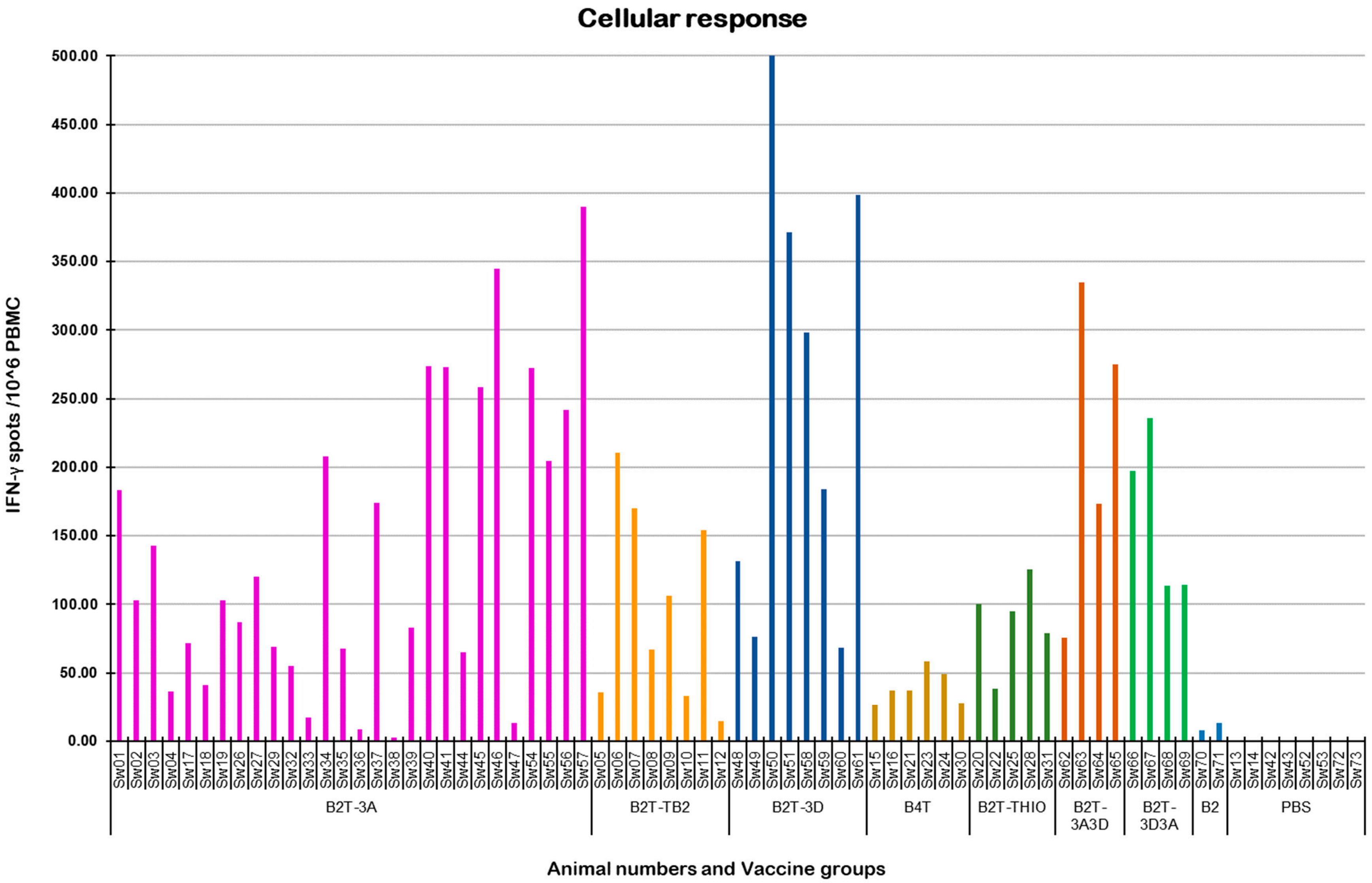

Figure S1. Antibody and cellular responses of 73 pigs to dendrimeric peptides. Antibody and cellular response values as in Table S2.
